# Comparative pathogenesis of Ebola virus and Reston virus infection in humanized mice

**DOI:** 10.1172/jci.insight.126070

**Published:** 2019-11-01

**Authors:** Beatriz Escudero-Pérez, Paula Ruibal, Monika Rottstegge, Anja Lüdtke, Julia R. Port, Kristin Hartmann, Sergio Gómez-Medina, Jürgen Müller-Guhl, Emily V. Nelson, Susanne Krasemann, Estefanía Rodríguez, César Muñoz-Fontela

**Affiliations:** 1 Bernhard Nocht Institute for Tropical Medicine, Hamburg, Germany.; 2 German Center for Infection Research (DZIF), Partner Site Hamburg, Hamburg, Germany.; 3 Institute for Neuropathology, University Medical Center Hamburg-Eppendorf, Hamburg, Germany.; 4 Heinrich Pette Institute, Leibniz Institute for Experimental Virology, Hamburg, Germany.

**Keywords:** Infectious disease, Virology, Mouse models

## Abstract

Filoviruses of the genus *Ebolavirus* include 6 species with marked differences in their ability to cause disease in humans. From the highly virulent Ebola virus to the seemingly nonpathogenic Reston virus, case fatality rates can range between 0% and 90%. In order to understand the molecular basis of these differences, it is imperative to establish disease models that recapitulate human disease as faithfully as possible. Nonhuman primates (NHPs) are the gold-standard models for filovirus pathogenesis, but comparative studies are skewed by the fact that Reston virus infection can be lethal for NHPs. Here we used HLA-A2–transgenic, NOD–*scid*–IL-2γ receptor–knockout (NSG-A2) mice reconstituted with human hematopoiesis to compare Ebola virus and Reston virus pathogenesis in a human-like environment. While markedly less pathogenic than Ebola virus, Reston virus killed 20% of infected mice, a finding that was linked to exacerbated inflammation and viral replication in the liver. In addition, the case fatality ratios of different *Ebolavirus* species in humans were recapitulated in the humanized mice. Our findings point to humanized mice as a putative model to test the pathogenicity of newly discovered filoviruses, and suggest that further investigations on Reston virus pathogenesis in humans are warranted.

## Abstract 2

Humanized mice reconstituted with human hematopoiesis recapitulate the pathogenicity of all ebolaviruses.

## Introduction

Filoviruses of the genus *Ebolavirus* comprise 6 known species. The most virulent for humans is Ebola virus (species *Zaire ebolavirus* [EBOV]), which has caused most of the outbreaks to date, including the West African epidemic of 2013–2016 (1) and the ongoing epidemic in the Democratic Republic of the Congo (DRC) (2). Two other members of the genus, Sudan virus (*Sudan ebolavirus* [SUDV]) and Bundibugyo virus (*Bundibugyo ebolavirus* [BDBV]), are also pathogenic for humans, with reported case fatality rates (CFRs) of 50% and 25%, respectively (3, 4). There is significantly less knowledge regarding the putative pathogenicity of Taï Forest virus (*Taï Forest ebolavirus* [TAFV]) and Reston virus (*Reston ebolavirus* [RESTV]) in humans. There is only one reported case of the former, a survivor (5, 6), and reports of seroconversion in the absence of disease for the latter (7, 8). The recent discovery of additional filoviruses and filovirus sequences in bats and other species (9–11) has underscored the need for animal models to test the putative pathogenicity of emerging filoviruses.

Nonhuman primates (NHPs), in particular rhesus and cynomolgus macaques, are the gold-standard models for the study of filovirus pathogenesis. Infection of NHPs with EBOV and SUDV reproduces many of the features of Ebola virus disease (EVD) in humans, and therefore, NHPs are preferred models for the development of vaccines and therapeutics (12, 13). However, this model presents limitations for comparative filovirus pathogenesis studies, since NHPs are also highly susceptible to RESTV and TAFV (14, 15).

We have previously shown that severely immune-compromised mice harboring human hematopoiesis are highly susceptible to EBOV infection (16). This model is based on the reconstitution of HLA-A2–transgenic NOD–*scid*–IL-2γ receptor–knockout mice with CD34^+^ human hematopoietic stem cells (HSCs) isolated from cord blood of HLA-matched donors (hereafter referred to as huNSG-A2 mice). Upon infection with WT EBOV (Mayinga variant), huNSG-A2 mice recapitulated many features of human disease including the incubation period, high lethality, the viremia, and high levels of serum aminotransferases (16).

Here we show that upon mucosal infection, EBOV was significantly more pathogenic than RESTV in huNSG-A2 mice. However, 20% of infected mice also died from RESTV infection. A comparative assessment of EBOV and RESTV pathogenesis indicated that lethal RESTV infection in this model was associated to exacerbated inflammation and sustained virus replication in the liver. These results suggest that under specific host conditions (e.g., immune suppression), RESTV may cause disease in humans. Moreover, the susceptibility of huNSG-A2 mice to viruses representative of different *Ebolavirus* species mimics that observed in humans, suggesting that mice harboring human immune components could serve as models to test the putative pathogenicity of newly discovered filoviruses.

## Results

### Mucosal RESTV replication kinetics is delayed with respect to that of EBOV.

**Figure 1:**
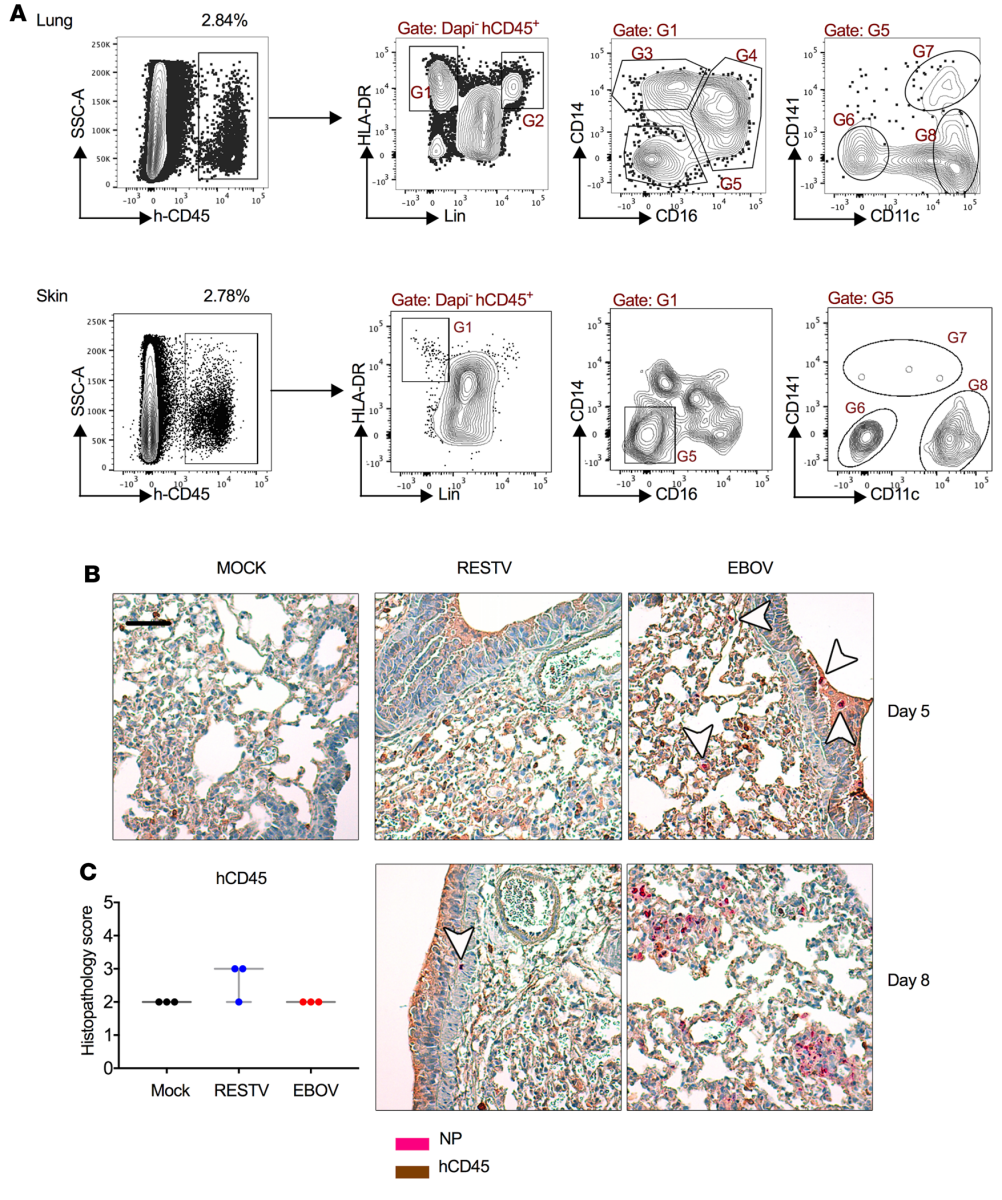
Mucosal exposure of huNSG-A2 mice to EBOV and RESTV. (**A**) Flow cytometry–based evaluation of the presence of mature human immune cells in skin (lower back area) and lung of huNSG-A2 mice. Gates indicate the percentage of cells expressing human CD45 (h-CD45) in either organ. The gating strategy in the right panels shows the presence of human antigen-presenting cells (APCs) (G1), B cells (G2), CD14^+^ monocytes (G3), CD16^+^ monocytes (G4), nonmonocytic APCs (G5), and human DC subsets (G6–G8). (**B**) Histopathological analysis of huNSG-A2 lung tissue after infection with EBOV or RESTV on the indicated days after infection. White arrowheads indicate the presence of infected cells, showing EBOV NP– and CD45-positive staining. Scale bar: 50 μm (**C**) Histopathology score (ordinal method, values of 0 to 5) assessing the levels of hCD45 staining in *n* = 3 lung sections of RESTV- and EBOV-infected and control (Mock) mice. Box-and-whisker plots represent minimum to maximum values. All scoring values are shown.

The natural portals of entry of ebolaviruses in humans are the skin and the mucosae (17). Therefore, we first evaluated the presence of human mature immune cells in the skin and mucosae of huNSG-A2 mice 12 weeks after transplantation of human CD34^+^ HSCs. Flow cytometry–based immunophenotyping showed that, indeed, mature antigen-presenting cells including human DCs and monocytes were observed in mouse lung and skin in the steady state (Figure 1A). In particular, the lung showed consistent reconstitution of human myeloid and lymphoid cell subsets, and thus we decided to use the intranasal route to mimic exposure to viruses via the respiratory mucosa.

We next performed an analysis of the infection kinetics of EBOV and RESTV in the respiratory mucosa in vivo. Histopathological analysis of lung samples using antibodies against human CD45 (hCD45), a pan-leukocyte marker, and the *Ebolavirus* nucleoprotein (NP), revealed stark differences in the replication kinetics of both viruses. On day 5 after infection, we already observed staining of EBOV NP in macrophage-like cells within the lung parenchyma, which colocalized with hCD45 (Figure 1B). On day 8 after infection, discrete clusters of EBOV replication were observed in the lung parenchyma. Conversely, replication of RESTV was significantly delayed and was not detectable prior to day 8 after infection (Figure 1B). These differences were not dependent on the levels of hCD45^+^ cells, which were comparable in RESTV- and EBOV-infected mice (Figure 1C).

These results are in agreement with RESTV having slower replication kinetics in cell culture than EBOV (18). The colocalization pattern observed also suggests that both viruses have a preference for human as opposed to mouse cells. Indeed, both viruses replicated to substantially higher titers in human macrophages and DCs compared with mouse macrophages and DCs (Supplemental Figure 1; supplemental material available online with this article; https://doi.org/10.1172/jci.insight.126070DS1). As expected, in these in vitro assays, RESTV replication was also slower than that of EBOV.

Taken together, our data suggested that mucosal exposure to EBOV and RESTV in mice reconstituted with human HSCs could serve as a model to study filovirus pathogenesis in a human-like environment and to dissect the mechanisms responsible for differences in filovirus pathogenicity.

### Infection of huNSG-A2 mice with ebolaviruses mimics species-specific CFRs.

**Figure 2:**
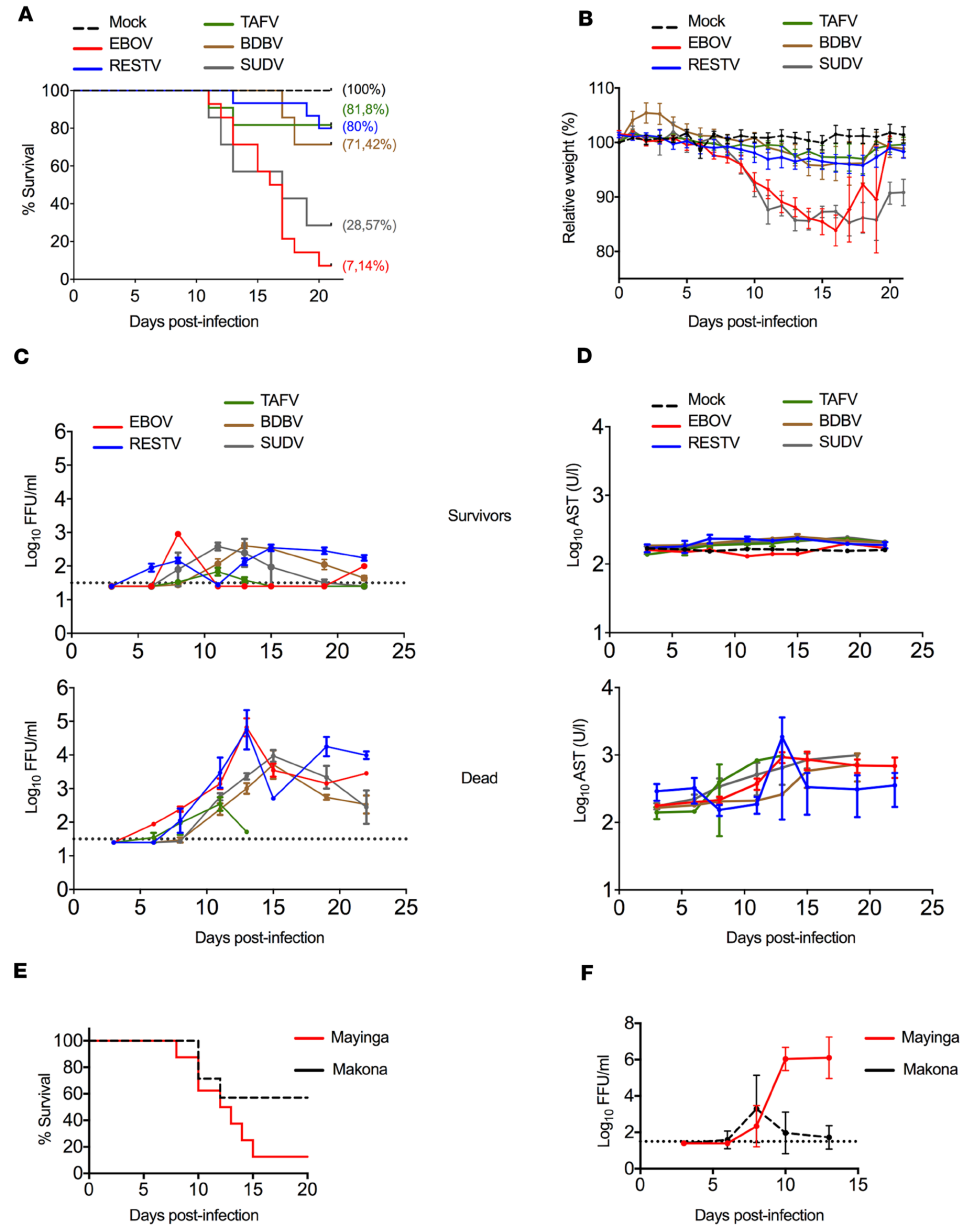
Comparative ebolavirus pathogenesis in huNSG-A2 mice. (**A**) Kaplan-Meier survival curves of infected mice. Mice were infected intranasally with 1000 FFU EBOV (*n* = 14), RESTV (*n* = 15), TAFV (*n* = 11), BDBV (*n* = 7), and SUDV (*n* = 7). Mock-infected mice (*n* = 11) received 20 μL PBS. Log-rank (Mantel-Cox) analysis indicated statistically significant differences between results for EBOV- and SUDV-infected mice and those for all the other groups (*P* < 0.0001). (**B**) Weight loss of infected huNSG-A2 mice. Nonparametric Kruskal-Wallis analysis followed by Dunn’s post hoc test analysis indicated significant differences between results for mice infected with EBOV (*P* = 0.025) and SUDV (*P* = 0.017) and those for the other groups. (**C**) Kinetics of viremia in surviving and nonsurviving mice from infection to experimental endpoint. Dotted lines represent the limit of detection of 50 FFU/mL. (**D**) Levels of AST in blood of infected mice (survivors and nonsurvivors). In the survivor group,the mock treatment group is represented by the black dashed line. (**E**) Survival curve of huNSG-A2 mice infected with the EBOV variants Mayinga (*n* = 8) and Makona (*n* = 7). Log-rank (Mantel-Cox) analysis indicated statistical significance (*P* = 0.042). (**F**) Kinetics of viremia from infection to experimental endpoint. Dotted lines represent the limit of detection of 50 FFU/mL. Throughout the figure, error bars represent mean ± SEM.

To determine whether infection of huNSG-A2 mice via mucosal exposure also recapitulated the lethality of other *Ebolaviruses* in humans, we sought to compare their susceptibility to 3 additional *Ebolavirus* species: SUDV, BDBV, and TAFV. Infection of huNSG-A2 mice with EBOV resulted in 92.86% lethality, comparable to that in our previous study using the intraperitoneal route (16). Similarly, infection with SUDV resulted in 71.43% lethality, which recapitulated the reported CFR of this virus in humans (3, 4) (Figure 2A). The high lethality of EBOV and SUDV was also associated with high morbidity (weight loss) in the model (Figure 2B). Infection with BDBV caused death in 28.58% of infected mice, which was also in agreement with the reported CFR in the only human outbreak described to date (19). TAFV infection killed 18.18% of infected mice, indicating that, while less pathogenic, TAFV may cause severe disease in our model. While significantly less pathogenic, RESTV infection resulted in death of 20% of infected mice, an outcome that was comparable to that of TAFV infection (Figure 2, A and B).

Among the main predictors of outcome of ebolavirus infection in humans are levels of viremia and serum aminotransferases (20–22). Thus, we compared the kinetics of viremia and AST in surviving and lethally infected huNSG-A2 mice. As expected, all surviving mice controlled viremia and maintained low levels of serum AST independently of the virus species to which they were exposed (Figure 2, C and D). Conversely, mice that died from infection had high levels of AST and virus in blood until death (Figure 2, C and D). An important difference was observed in the case of mice infected with TAFV. Mice that died from TAFV infection showed high levels of circulating AST (Figure 2D); however, the overall level of TAFV in blood was significantly lower than that observed for the other ebolaviruses (Figure 2C). Apart from its reported high lethality in chimpanzees (15, 23), little is known about TAFV pathogenesis in humans and NHPs. Our model suggests that at least the kinetics of replication of this virus differs from that of other species in the genus.

Previous reports have indicated differences in pathogenesis between the Mayinga and Makona variants of EBOV, in humans (21, 24) as well as NHPs and immunodeficient mice (25, 26). Thus, we next determined whether our model would also recapitulate differences in pathogenesis between these 2 EBOV variants. Indeed, while EBOV Mayinga was highly lethal in huNSG-A2 mice and caused high levels of viremia, mice infected with EBOV Makona showed reduced lethality (50%), as well as reduced levels of virus in blood (Figure 2, E and F).

In summary, our data indicated that upon mucosal exposure, huNSG-A2 mice showed distinct susceptibility to different *Ebolavirus* species and EBOV variants, suggesting that this model could provide insight into virus species–specific pathogenicity mechanisms and the putative virulence of newly discovered filoviruses.

### The lethality of RESTV in humanized mice is associated with high inflammation.

In order to investigate why RESTV infection was lethal for a subset of mice, we first performed Luminex-based analysis of cytokine production in RESTV- and EBOV-infected mice over the course of infection. To determine the specific contribution of the human immune system in our model, we utilized panels to assess the expression of human cytokines, chemokines, and coagulation markers.

**Figure 3:**
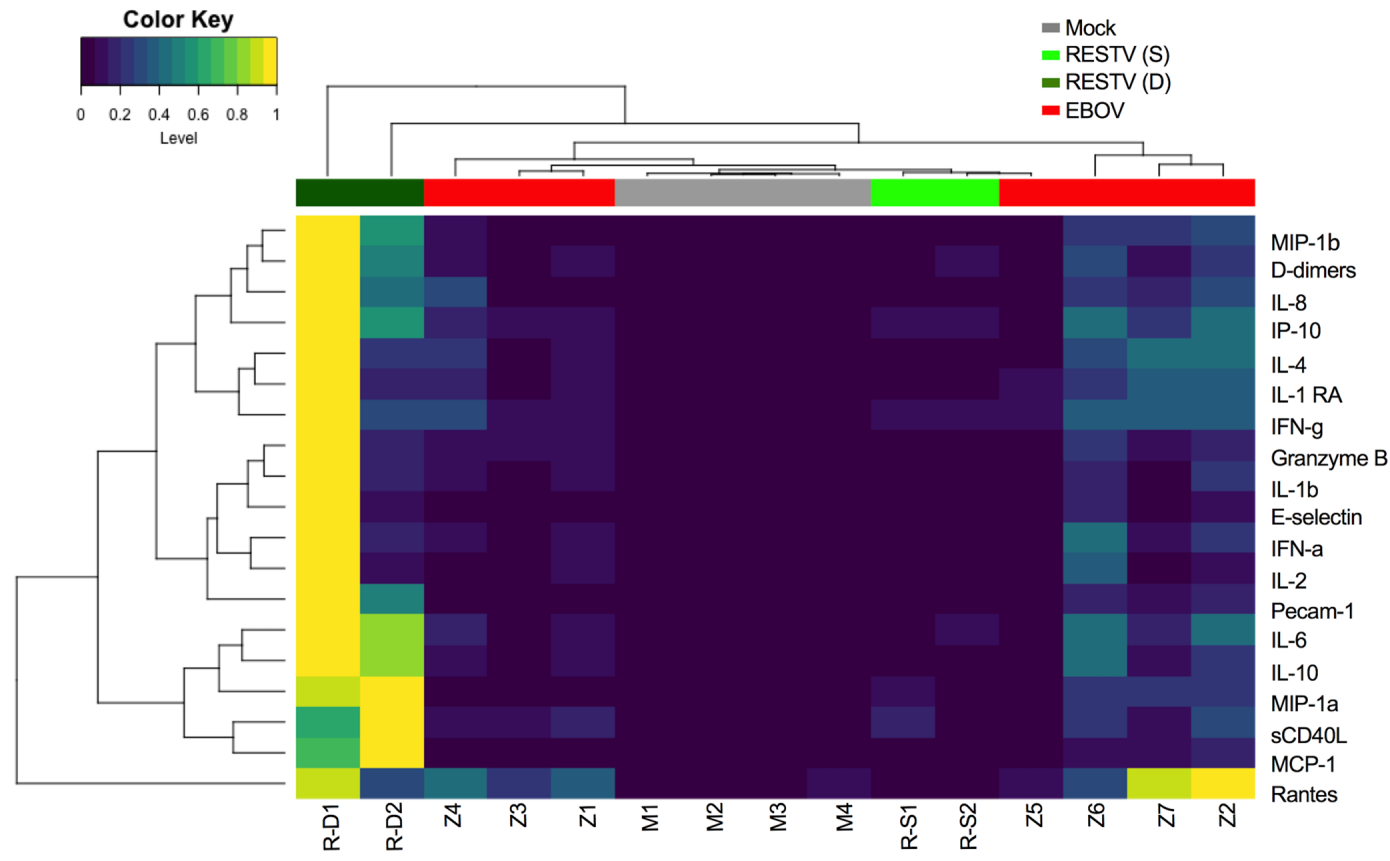
Inflammatory profile of mice infected with EBOV (Mayinga variant) and RESTV. Heatmap showing levels of the indicated analytes in plasma of mice infected with either EBOV (Z, *n* = 7) or RESTV (R-D, dead; R-S, survivor; *n* = 4) on day 3 after infection. Hierarchical clustering of samples was performed based on euclidean distance using complete linkage. Cytokine data collected via Luminex multiplexed ELISA assays were normalized using min-max normalization, which normalized cytokine values between 1 and 0. Mock-infected mice (M) that received PBS are shown as controls. Visualization of cytokine profiles was performed using the R function heatmap.2 implementing euclidean distance and using ward.D clustering.

Expression of high levels of proinflammatory cytokines at early time points after infection predicted fatal outcome in mice infected with either EBOV (Mayinga) or RESTV. Thus, on day 3 after infection, we observed high levels of proinflammatory chemokines (e.g., macrophage inflammatory proteins–1α [MIP-1α], MIP-1β, IL-8, IP-10), IFNs, markers of endothelial dysfunction (E-selectin, Pecam-1), and coagulation markers (e.g., D-dimers) in mice that died from either EBOV or RESTV infection (Figure 3A). Levels of proinflammatory cytokines in serum were, however, significantly higher in mice that died from RESTV infection and were maintained until day 10 after inoculation, indicating sustained inflammation over the course of infection (Supplemental Figure 2). Overall, the highest levels of proinflammatory mediators were observed in mice with high levels of viremia, elevated levels of serum AST, and short time to death after exposure (Table 1). These data strongly suggest that the severity of EBOV infection in huNSG-A2 mice can be monitored using the same biomarkers proposed for humans, namely, levels of viremia and inflammation. Our results also indicated that death from either EBOV or RESTV infection was associated with high levels of expression of proinflammatory cytokines and elevated levels of serum aminotransferases, suggesting liver damage.

### The lethality of RESTV in humanized mice is associated with high levels of virus replication in the liver.

**Figure 4:**
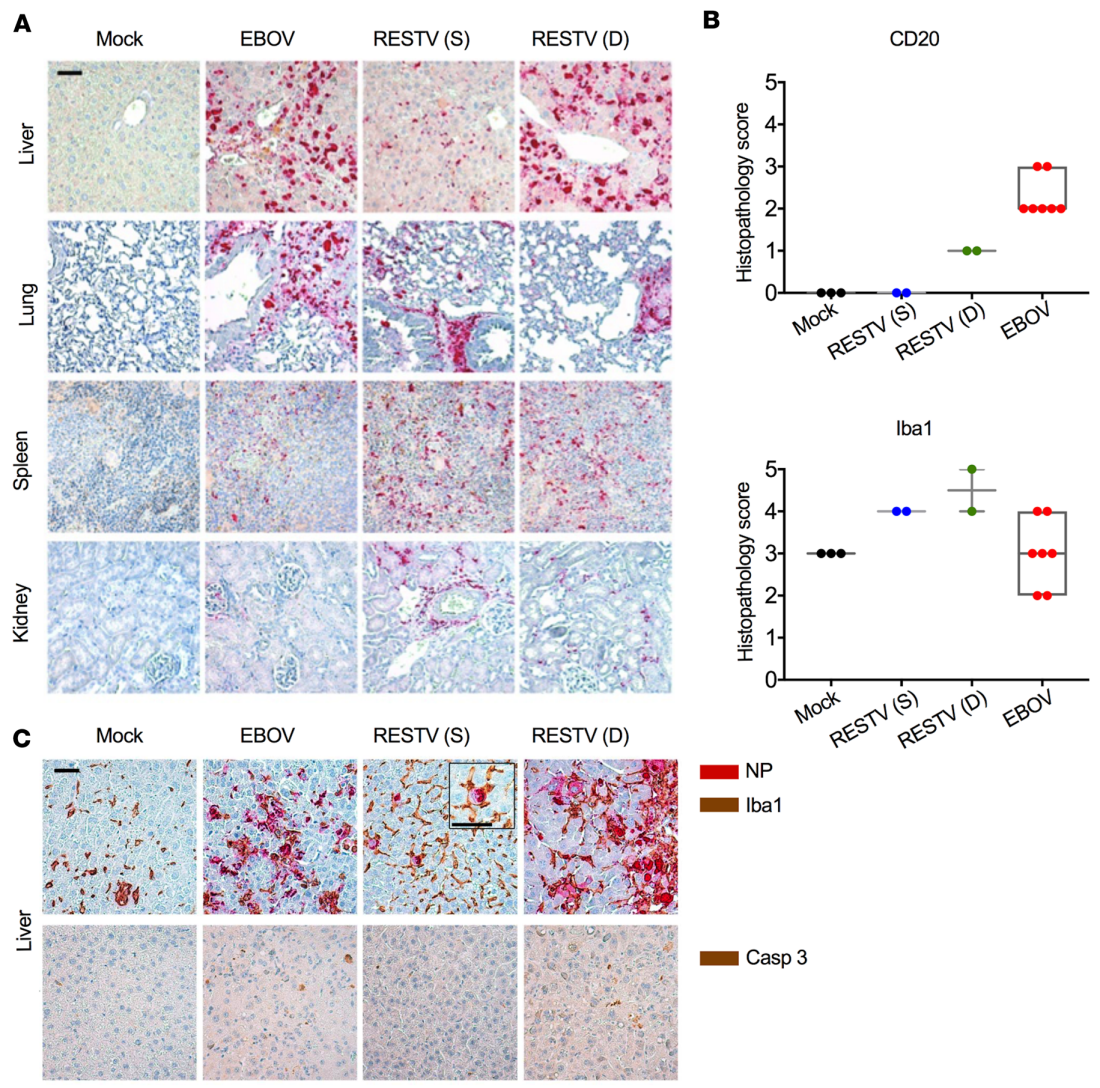
Liver pathology of EBOV- and RESTV-infected mice. (**A**) Histopathological findings in tissue sections of huNSG-A2 mice infected with EBOV and RESTV. In lethally infected mice, pathological assessment was done at the time of death (necropsy); surviving mice were euthanized on day 30 after infection. Red indicates staining of EBOV and RESTV NP in the indicated tissues. (**B**) Histopathological score (ordinal method, values of 0 to 5) for human CD20- and Iba1-positive cells in liver sections of mock-infected (*n* = 3), RESTV-infected/surviving (S) (*n* = 2), lethally RESTV-infected (D) (*n* = 2), and EBOV-infected (*n* = 7) mice. Box-and-whisker plots represent minimum to maximum values. All scoring values are shown. (**C**) Histopathological analysis of liver sections subjected to immunohistochemistry staining with anti-NP (red), anti-Iba1 (brown) and anti–caspase-3 (Casp 3; brown) antibodies. The magnified image inside the square shows an infected macrophage surrounded by Iba1^+^ cells. Scale bars: 50 μm. Mock-infected mice received 20 μL PBS.

To further assess the relationship among human immune cell function, virus replication, and liver damage, we performed histopathological analysis of tissues during necropsies of RESTV-infected and EBOV-infected mice. Interestingly, while histopathology revealed stark differences between mice that survived RESTV infection and those that died, these differences were restricted to the liver. Mice infected with RESTV showed similar levels of virus replication in spleen and lung, which was independent of the outcome. Mice that survived RESTV showed even higher levels of virus replication in the kidney than their lethally infected counterparts. However, mice that died from EBOV or RESTV infection showed significantly higher levels of virus replication in the liver, whereas RESTV-surviving mice controlled virus replication in this organ (Figure 4A). These high levels of virus replication in lethally infected mice (EBOV and RESTV) correlated with high levels of infiltration in the liver of immune cells including human B lymphocytes and macrophages/monocytes (Figure 4B). Of note, in RESTV survivors, we observed active phagocytosis of infected RESTV NP^+^ cells by Iba1^+^ monocytes/macrophages (Figure 4C, inset). Although indirectly, these findings strongly suggest that virus replication in these mice may be associated with infiltration of myeloid cells that support virus replication. Lethality caused by either EBOV or RESTV was also associated with higher levels of caspase-3 staining in the tissue sections (Figure 4C). These results indicated that fatal EBOV and RESTV infection in huNSG-A2 mice was characterized by higher levels of virus replication in the liver, infiltration of immune cells, and increased levels of apoptosis.

## Discussion

In this study, we present a mouse model with mature human peripheral immune cells that is susceptible to infection with nonadapted ebolaviruses. We utilized intranasal inoculation of virus in order to mimic mucosal exposure, a probable route of infection during human EVD outbreaks (27, 28). Recently, pathogenesis studies using the mucosal route of exposure to EBOV have also been conducted in NHPs and guinea pigs. In both models, intranasal inoculation of EBOV resulted in delayed time to death compared with intramuscular inoculation (29, 30). In another NHP study, oral and conjunctival exposure to low doses of EBOV resulted in survival (31). These findings strongly suggest that mucosal immunity may play an important role in precluding systemic dissemination of EBOV from the initial points of infection. However, in the humanized mouse model utilized in our study, we did not observe differences in lethality or time to death after infection when comparing intranasal and intraperitoneal inoculation of virus (16). These findings probably reflect limitations in the immune responses in our model, such as defects in immune cell migration to tissue-draining lymph nodes and poor CD4^+^ T cell help.

NSG mice transplanted with human CD34^+^ HSC have been previously shown to mount human-like immune responses to viral infections, including those caused by HIV, Ebola virus, dengue virus, and adenovirus, among others (32–35). Myeloid and T cell activation as well as production of human immunoglobulins have been described in humanized NSG mice. In the HLA-A2–transgenic model, virus-specific CD8^+^ T cell responses have been also demonstrated (34, 35), although in the case of experimental dengue infection, they resulted in inefficient virus clearance (35). In our study, however, we observed that mice surviving infection were able to control virus loads and, in some cases (i.e., SUDV and BDBV) clear viremia. We hypothesize that such control is probably dependent to a great extent on HLA-A2–restricted CD8^+^ T cells similarly to what has been previously described for acute adenovirus infection in the same model (34). This would be also consistent with our previous observation that nontransplanted NSG-A2 mice experience a lengthy chronic disease characterized by sustained viremia (16). Although further experiments are needed to test this hypothesis, this would be also in line with the important role of CD8^+^ T cell–mediated immunity in recovery from acute EVD (24). However, as mentioned above, there are also several limitations of the NSG model, including poor CD4^+^ T cell–dependent responses, long-term problems associated to graft-versus-host disease, hemophagocytic lymphohistocytosis, and lack of immunoglobulin isotype switching (36, 37). Despite the latter, huNSG-A2 mice that died from EBOV or RESTV infection showed evidence of recruitment of human macrophages and B cells to the sites of infection, in particular to the liver. These results are consistent with the massive generation of activated CD20^+^ B cells after EBOV infection in humans (38) and their recruitment to infection sites (39).

In our study we also observed that lethality associated with either EBOV or RESTV infection was associated with early and sustained production of proinflammatory cytokines, high levels of viremia, and high levels of AST. These findings are in agreement with data collected during human outbreaks that indicated cycle threshold (Ct) values (as a surrogate of viremia) and inflammation as biomarkers with predictive potential (20, 22, 40). Interestingly, mice that died from infection with either virus also showed early upregulation of D-dimers, which suggested that fatal ebolavirus infection in humanized mice is also associated with coagulopathy. This observation is in line with the presence of focal bleeding in necropsies of EBOV-infected huNSG-A2 mice (16).

The reasons for the stark differences in EBOV and RESTV pathogenicity are unknown. Several factors have been proposed, including the ability of the virus glycoprotein ability to direct entry into target cells (41) and perhaps the efficacy of viral protein 35 (VP35) and VP24 to counteract the type I IFN response in infected cells (42, 43). Presumably for these reasons, RESTV replication in cell culture is delayed with respect to that of EBOV (18). In this study using mice harboring human peripheral immune cells, we have shown that these differences in replication kinetics are also present in vivo, and that both EBOV and RESTV have a preference for human cells. Our findings are consistent with recently published data demonstrating that lethal EBOV infection and nonlethal RESTV infection are characterized by substantial differences in virus replication in the liver (44) and strongly support the notion that liver pathology is a central feature of lethal filovirus infection (21, 45, 46). However, our data also indicate that under circumstances whereby RESTV is able to colonize the liver, this may lead to lethal infection. We hypothesize that the mucosal barrier may be of great importance for controlling early RESTV replication. However, this barrier can be overcome, leading to virus dissemination, high levels of inflammation, coagulopathy, and high levels of virus replication in the liver.

Finally, we propose that the model presented here may be of use to test the putative pathogenicity of newly discovered filoviruses. Even though further studies are needed to dissect the pathogenic features of ebolaviruses in huNSG-A2 mice, our initial studies indicate that the susceptibility of these mice to specific ebolaviruses was very similar to that of humans. Moreover, within the *Zaire ebolavirus* species, huNSG-A2 mice were significantly less susceptible to Makona virus compared with Mayinga virus, which was in agreement with reported CFRs in human (17, 21) and animal model studies (25, 26).

## Methods

### Generation of huNSG-A2 mice.

Humanized mice were generated as previously described (16). Briefly, CD34^+^ human HSCs were positively selected (EasySep Human CD34 Positive Selection Kit, StemCell Technologies) from cord blood. To reconstitute the human hematopoietic system in NSG-A2 mice, we utilized the NOD.Cg-*Prkdc^scid^* *Il2rg^tm1Wjl^* Tg(HLA-A2.1)1Enge/SzJ mouse strain purchased from the Jackson Laboratory. All experiments were conducted with 5-week-old NSG-A2 (HLA-A2.1) females conditioned by sublethal irradiation (240 cGy). Four hours after irradiation, mice underwent intravenous (retro-orbital) transplantation of 10^6^ HLA-A2–matching CD34^+^ HSCs per mouse. Eight weeks after HSC transplantation, blood samples were collected, and the presence of human hematopoietic cells was quantified by flow cytometry using anti–human CD45 antibody BV510 (clone HI30; 304035, BioLegend) and anti–mouse CD45.2 antibody PerCP/Cy5.5 (clone 104; 109825, BioLegend). Samples were analyzed in a LSR II Fortessa flow cytometer (BD Biosciences). Mice containing 30%–50% HSPCs were selected for all the experiments. All infection experiments were performed at week 9–10 after engraftment.

### Mouse infection and disease monitoring.

Mice were intranasally infected with 1000 focus-forming units (FFU) of EBOV (Ebola virus/H.sapiens-tc/COD/1976/Yambuku-Mayinga and Ebola virus/H.sapiens-wt/GIN/2014/Makona-C07). RESTV (Pennsylvania strain) and TAFV (Tai Forest virus/H.sapiens-tc/CIV/1994/Pauleoula-CI) were donated by Stephan Becker (Philipps University of Marburg,Marburg, Germany). BDBV (Bundibugyo virus/H.sapiens-tc/UGA/2007/Bundibugyo-200706291) and SUDV (Sudan virus/H.sapiens-tc/UGA/2000/Gulu-808892) were donated by Christina Spiropoulou (Centers for Disease Control and Prevention, Atlanta, Georgia, USA). All virus stocks were tested for mycoplasma (MycoAlert Mycoplasma Detection Kit, Lonza) and certified mycoplasma free.

A mock treatment group of mice received PBS and was kept as a negative control. All mice were kept in individually ventilated cages inside a biosafety level 4 (BSL4) laboratory at the Bernhard Nocht Institute and fed autoclaved food and water. Mice were monitored (weight, temperature, and general body scoring) daily over the course of the disease. According to the guidelines approved for our study, animals with severe signs of disease such as bleeding, lethargy, temperature lower than 28°C, or weight loss of more than 20% of their original weight were euthanized.

### Immunofocus assay.

Infectious virus particles in blood and organ samples were determined by immunofocus assay. Organs were weighted and homogenized in tubes containing 1 mL DMEM using Lysing Matrix D tubes (MP Biomedicals) containing a bead mill. Vero 81 (ATCC CCL-81) cells seeded in 24-well plates were incubated with 200 μL of serial 10-fold dilutions of homogenized organs and blood samples. The inoculum was removed after 1 hour and replaced with a 1% methylcellulose medium overlay. After 6 days of incubation, overlay was removed, and cells were fixed with 4% formaldehyde, washed with water, and permeabilized with 0.5% Triton X-100 in PBS. After washing with PBS and blocking with 5% FCS in PBS, cell foci were detected using an in-house-generated mouse polyclonal anti–pan-Ebolavirus NP primary antibody (1:2000 overnight at 4°C). For detection of foci, cells were incubated with secondary peroxidase-conjugated AffiniPure Sheep Anti-Mouse IgG (H+L) antibody (515-035-003, Jackson ImmunoResearch Laboratories Inc.) at a 1:40,000 dilution for 45 minutes at room temperature. After washing with water, foci were visualized with tetramethylbenzidine (TMB) and counted.

### Replication assay.

PBMCs were isolated from the blood of healthy donors by centrifugation in a lymphocyte separation medium (Ficoll). The recovered monocyte fraction was depleted of cell contaminants by CD14^+^ selection (Miltenyi Biotec), ensuring purification rates of ≥95%. Immature DCs and monocyte-derived macrophages were obtained upon incubation of cells in complete RPMI-1640 (Life Technologies) supplemented with 200 mM L-glutamine (Life Technologies), 10 mM HEPES buffer (Life Technologies), 10% decomplemented FCS, and 8 μg/mL Gentamicin (Life Technologies) for 5 days with 50 ng/mL GM-CSF (PeproTech) and 50 ng/mL IL-4 (PeproTech) or 40 ng/mL M-CSF (PeproTech), respectively. Monocyte-derived human DCs and macrophages were then infected at an MOI of 0.5 as described above.

For generation of murine DCs and macrophages, murine bone marrow was extracted from 8-week-old male WT C57BL/6 mice (The Jackson Laboratory). Briefly, mice were euthanized, and bone marrow from tibiae and femurs was harvested by cutting the edges of the bones and flushing with a 29G needle in sterile conditions. Then, red blood cells were lysed (BD Pharm Lysing Buffer), and cells were seeded at a density of 10^6^ cells/mL in 24-well plates. For differentiation into murine DCs, cell culture medium (RPMI with 10% FCS) was supplemented with 50 ng/mL GM-CSF (PeproTech). For differentiation of bone marrow progenitors into macrophages, the medium was supplemented with 20 ng/mL M-CSF (Miltenyi Biotec). Each cell type was then infected in triplicate at an MOI of 0.01 with EBOV or RESTV or with 2.5% FCS supplemented RPMI as mock control. Infectious viral particles were quantified daily in tissue culture supernatants using immunofocus assays as described above.

### Clinical chemistry.

Serum samples from infected mice were diluted 1:10 in water, and quantification of serum aminotransferases (AST) was determined by using commercially available GOT/AST Fuji DRI-CHEM slides in Fujifilm in a DRI-CHEM NX500 analyzer. The limit of detection for AST was 10 U/L.

### Flow cytometry.

Single-cell suspensions were prepared from organs by cutting tissues into small fragments, followed by enzymatic digestion for 30 minutes at 37°C with Collagenase D (2 mg/mL) (Roche) and DNAase (50 μg/mL) in RPMI-1640 medium. Tissue fragments were further disrupted by passage through a 70-μm nylon cell strainer (BD Biosciences). Red blood cells were lysed with BD Pharm Lyse buffer (BD Biosciences). Cells were then stained for viability with Zombie NIR (BioLegend) for 30 minutes at room temperature, washed twice with PBS, and blocked with Human TruStain FcX (Fc receptor blocking solution, BioLegend) for 20 minutes at room temperature. Cells were stained with an antibody cocktail consisting on the following multiparametric flow cytometry panel: CD45-FITC (2D1; 368507, BioLegend), CD1c-APC (L161; 331523, BioLegend), CD38–PerCP-Cy5.5 (HIT2; 303521, BioLegend), CD16-PECy7 (3G8; 302015, BioLegend), CD141-PE (M80; 344103, BioLegend), HLADR-BV785 (L243; 307641, BioLegend), CD8-BV570 (RPA-T8; 301037, BioLegend), CD14-BV510 (M5E2; 301841, BioLegend), CD56 (HCD56; 318325, BioLegend), CD19-PB (HIB19; 982404 BioLegend), CD4-BUV737 (SK3; 564305, BD Biosciences), and CD3-BV650 (OKT3; 317323, BioLegend). Formaldehyde-inactivated samples were acquired using an LSRFortessa (BD Biosciences) and analyzed with FlowJo software.

### Histology and immunohistochemistry.

Mouse tissue samples were fixed in 4% formalin/PBS and were embedded in paraffin. Sections were then stained with H&E or processed for immunohistochemistry as follows: After dewaxing and inactivation of endogenous peroxidases (PBS/3% hydrogen peroxide), antibody-specific antigen retrieval was performed. Sections were blocked (PBS/10% FCS) and incubated with primary antibodies for rat anti–mouse CD3 (T cells; OKT; 317301, BioLegend), anti-CD20 (B cells; Dako Omnis clone L26; GA604), anti–cleaved caspase-3 (apoptosis marker; ab2302, Abcam), anti-CD14 (monocytes; ab183322, Abcam), or anti–Ebola-NP (clone KZ51 IgG1 mouse; Absolute Antibody). Bound primary antibodies were detected with anti-mouse, anti-rabbit or anti-rat *N*-Histofine Simple Stain MAX PO immune-enzyme polymer (Nichirei Biosciences Inc.) and stained with DAB substrate using the ultraView Universal DAB Detection Kit (Ventana). Tissues were counterstained with hematoxylin.

### Luminex multiplex assay.

Sera from all mice were collected on days 3, 6, 9, 12, 15, 18, 21, and 23 after infection, and analysis of cytokine concentrations was performed using ProcartaPlex Multiplex Immunoassay Magnetic Bead Panel Premix 19 Plex (PPX-19) according to the manufacturer’s instructions. A Luminex 200 system (Millipore) was used for data acquisition.

### Heatmaps.

Visualization of cytokine profiles was performed using the R function heatmap.2, implementing euclidian distance and using the ward.D clustering method to generate the dendrograms. Min-max normalization was performed for each cytokine to normalize data ranges between 1 and 0.

### Statistics.

Statistical analyses were done using Graphpad Prism 6 software. All data are presented as mean ± SEM. For multiple comparisons, nonparametric 1-way ANOVA on ranks (Kruskal-Wallis test) followed by Dunn’s post hoc test was used. For comparison of survival curves, log-rank (Mantel-Cox) tests were used. A *P* value less than 0.05 was considered significant.

### Study approval.

Human HSCs were purified from cord blood obtained at the Asklepios Klinic Nord in Hamburg. All patients agreed to donation of biological material by informed written consent under a protocol approved by the Ethics Commision of the Medical Association of Hamburg (WF-054/15). Animal experiments were performed under protocols approved by the German animal protection authorities (Behörde für Gesundheit und Verbraucherschutz, Hamburg, approval 110/17). All infection experiments were carried out in the BSL4 laboratory of the Bernhard Nocht Institute by experienced and highly trained personnel using positive pressure biocontainment suits.

## Author contributions

BEP and CMF designed the experiments. BEP, PR, MR, AL, EVN, and JRP performed experiments. KH and SK performed histopathology experiments and analyzed the data. SGM, JMG, and ER generated humanized mice for the study. ER and CMF coordinated the study. BEP and CMF wrote the manuscript.

## Supplementary Material

Supplemental data

## Figures and Tables

**Table 1:**
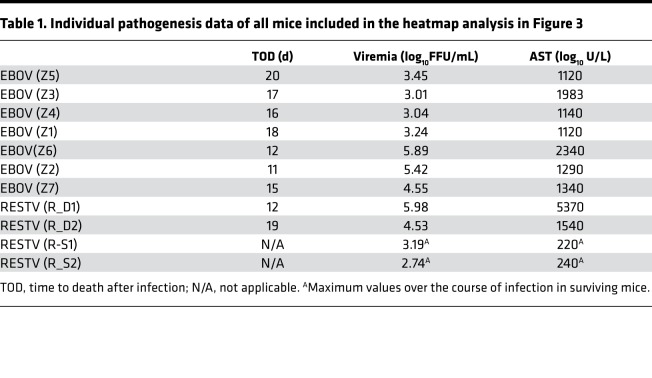
Individual pathogenesis data of all mice included in the heatmap analysis in Figure 3

## Version 1 09/24/2019

In-Press Preview

## Version 2 11/01/2019

Electronic publication
