# Supplementary material for: Comparative pathogenesis of Ebola virus and Reston virus infection in humanized mice
Source: JCI Insight. 2019 Nov 1;4(21):e126070. doi: 10.1172/jci.insight.126070 (PMC6948759; doi:10.1172/jci.insight.126070)
Supplement: Supplemental data [file jciinsight-4-126070-s006.pdf]

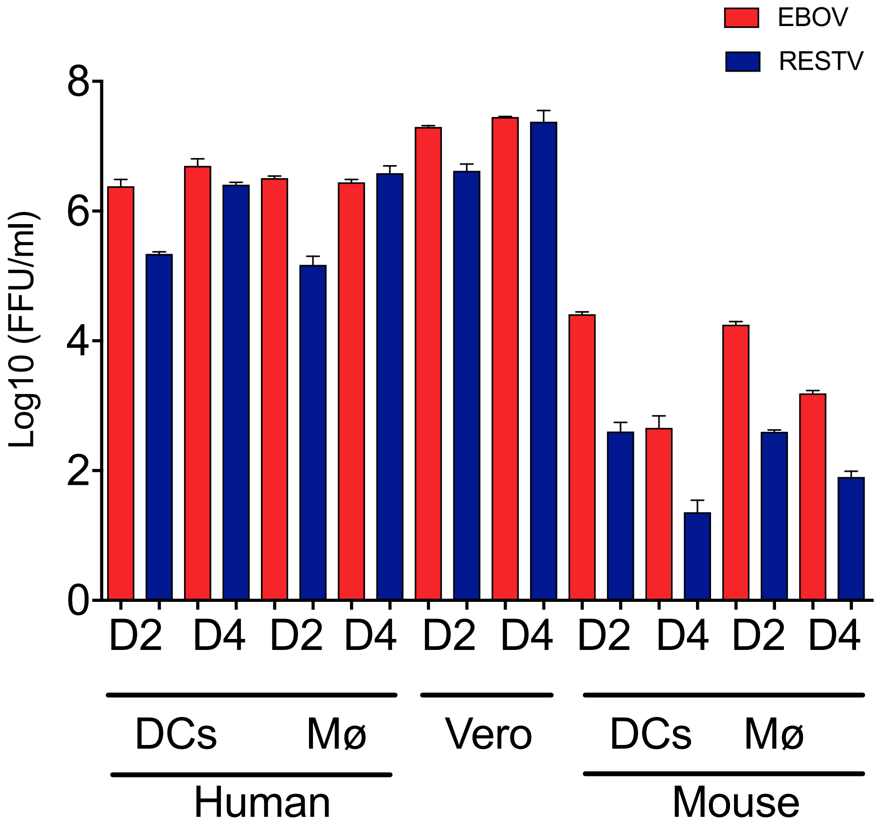

Supplemental Figure 1. Growth kinetics of EBOV and RESTV in vitro. Human and mouse dendritic cells (DCs) and macrophages (Mø) were infected with RESTV or EBOV at an MOI of 1. Cell supernatants were collected at the indicated days post-infection (D). Virus titers were evaluated by focus-formation assays.

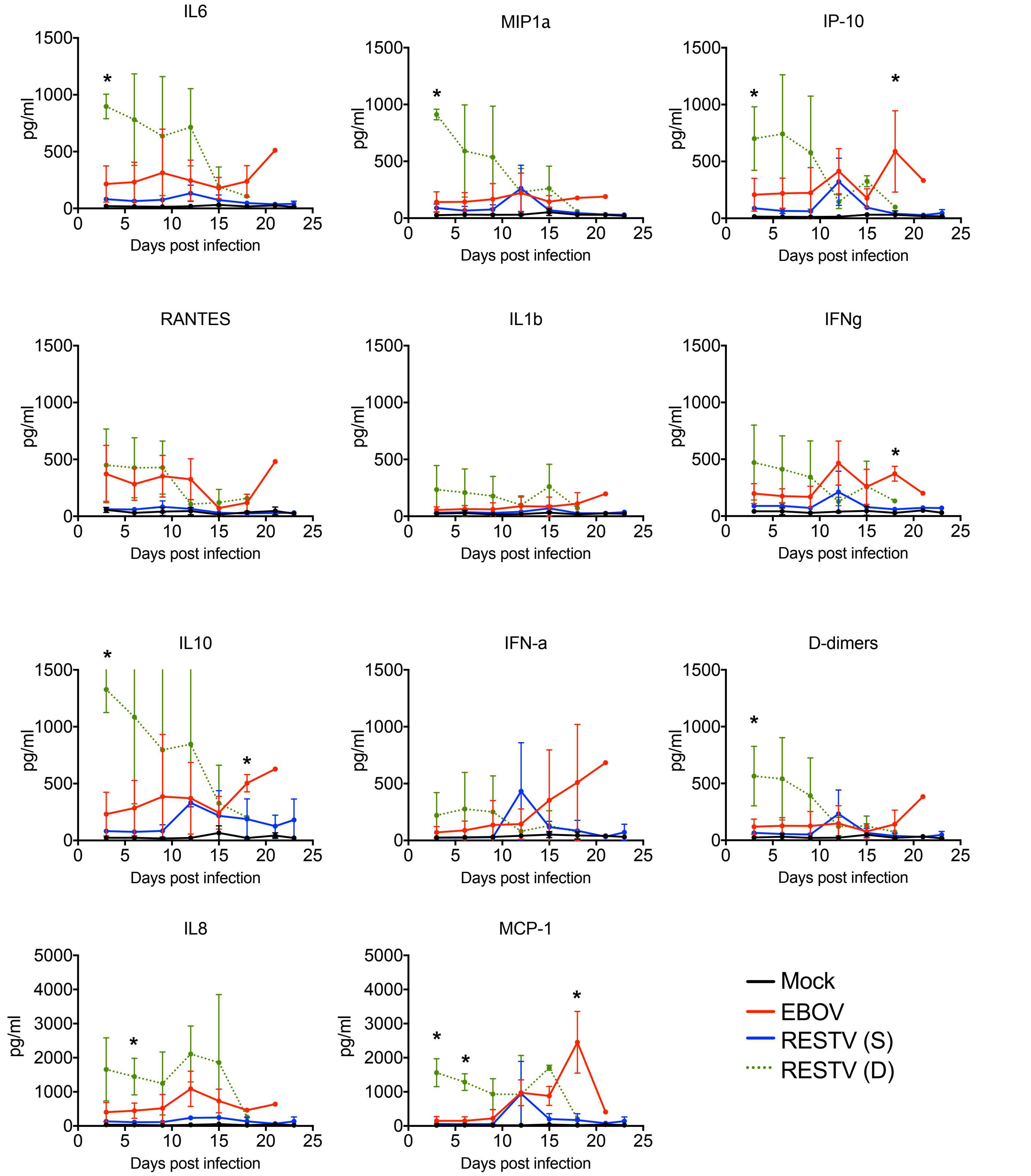

Suplemental Figure 2. Longitudinal evaluation of cytokine expression in EBOV (Mayinga variant) and RESTV infected mice. Asterisks (*) indicate time points where the differences were statistically significant as determined by Two-way ANOVA followed by Bonferroni's post-test.
